# 

**Published:** 1886-06

**Authors:** 


### JUNE, 1886.

### CONTENTS.

Page.

ORIGINAL PAPER:—

Removal of the Uterine Appendages. By J. GREIG SMITH ... ... 73

CLINICAL RECORDS:—

The Vomiting of Pregnancy. By JOHN MEREDITH, M.D. Edin. ... 95

Some Unusual Symptoms in a case of Hydrocephalus. By W. BARRETT ROUÉ, M.D., M.S., F.R.A.S. ... ... ... ... ... ... ... ... ... ... 102

Case of Typhlitis. By J. G. SWAYNE, M.D. Lond. ... ... ... ... 108

Notes on a case of Bronchial Croup. By G. ARTHUR BROWN. ... 115

PERISCOPE OF MEDICAL AND SURGICAL PROGRESS:—

Notes on Practical Surgery. By W. J. PENNY, F.R.C.S. ... ... ... 119

Notes on Ocular Therapeutics. By F. RICHARDSON CROSS, M.B. Lond., F.R.C.S. ... ... ... ... ... ... ... ... ... ... ... ... ... ... 127

Microzymes in Nutrition ... ... ... ... ... ... ... ... ... ... 133

REVIEWS AND NOTICES OF BOOKS ... ... ... ... ... ... ... ... ... 137

SCRAPS ... ... ... ... ... ... ... ... ... ... ... ... ... ... ... ... 143

